# Time to next pregnancy in spontaneous pregnancies versus treatment cycles in fertile patients with recurrent pregnancy loss

**DOI:** 10.1186/2054-7099-1-5

**Published:** 2015-04-21

**Authors:** Candice O Perfetto, Gayathree Murugappan, Ruth B Lathi

**Affiliations:** Division of Reproductive Endocrinology and Infertility, Department of Obstetrics and Gynecology, Stanford University Medical Center, 900 Welch Rd, Suite 15, Palo Alto, CA USA 94304

**Keywords:** Recurrent pregnancy loss, Time to pregnancy, Spontaneous pregnancy, Time to next pregnancy in spontaneous pregnancies versus treatment cycles in fertile patients with recurrent pregnancy loss

## Abstract

**Background:**

The current standard of care for management of patients with recurrent pregnancy loss is expectant management. However, the emotional impact of pregnancy losses and the urgency to conceive often leads couples to consider a variety of fertility treatments. The objective of this study is to report the time to next pregnancy and subsequent live birth and miscarriage rates in fertile patients with recurrent pregnancy loss (RPL) who choose to attempt spontaneous conception compared to those that opt to pursue fertility treatment.

**Methods:**

Retrospective cohort study of one hundred and fifty-eight fertile RPL patients treated at a university-based fertility center. Patients were followed for a minimum of 6 months. Patients were encouraged to attempt spontaneous conception, but allowed to initiate fertility treatments (ovarian stimulation, insemination, IVF or PGS) according to their preferences. Main outcome measures were time to next pregnancy and pregnancy outcome.

**Results:**

For those patients who achieved a spontaneous conception, 88% conceived within 6 months, with a median time of 2 months and range of 1–10 months. Patients using IUI, IVF and PGS conceived in a median of 3, 4 and 5 months, respectively. The live birth rate and clinical miscarriage rate was not improved with any fertility treatment.

**Conclusions:**

In the fertile RPL patient population, there does not appear to be a benefit to proceeding directly with fertility treatment. Patients should be encouraged to attempt spontaneous conception for at least 6 months.

**Electronic supplementary material:**

The online version of this article (doi:10.1186/2054-7099-1-5) contains supplementary material, which is available to authorized users.

## Background

Recurrent pregnancy loss (RPL) is a condition that affects up to 2-5% of couples attempting conception [1]. After a thorough work-up, including parental karyotypes, anti-phospholipid antibodies and a uterine cavity evaluation, almost 50% of patients have no diagnosis for their recurrent early miscarriages [2, 3]. Recommended treatments for unexplained RPL include nutritional optimization, emotional support, close surveillance and ultimately rely on spontaneous conception [4, 5]. Live birth rates with preconception counseling and expectant management is >60% for most patients, dependent on maternal age and the number of prior losses [6].

The emotional impact of RPL and the urgency to conceive often leads couples to consider a variety of fertility treatments, such as intrauterine insemination (IUI), controlled ovarian hyperstimulation (COH) and in vitro fertilization (IVF), to reduce the amount of time to their next pregnancy, as well as increase their chance of a pregnancy or live birth sooner. Possible benefits of undergoing fertility treatments are increased number of mature oocytes available per cycle, improved luteal phase support, improved timing of fertilization and aneuploidy screening in the setting of pre-implantation genetic screening (PGS) [7]. Benefits have to be balanced against the potential negative effects of fertility treatments, including but not limited to financial costs, multiple gestation and ovarian hyperstimulation syndrome.

Although some researchers have reported favorable live birth outcomes at 5, 10 and 15 years after the initial RPL consultation, most patients do not consider 5 years a reasonable amount of time to achieve a live birth [8]. In fact, at the initial visit, most patients desire a more specific time line on how long it typically takes for a fertile patient with RPL to get pregnant spontaneously, compared to initiating a fertility treatment immediately. To determine which option is best for these patients, we sought to evaluate the time to next pregnancy and subsequent miscarriage rates in fertile patients with RPL who choose to attempt spontaneous conception compared to those that opt to pursue fertility treatment immediately.

## Methods

This is a retrospective cohort study of patients seen in an RPL clinic at a University-based fertility center from 2010 to 2013. Institutional review board approval was obtained for this study. Patients with a history of 2 or more clinical miscarriages (CM) underwent an RPL work-up recommended by the American Society for Reproductive Medicine, including blood work for parental karyotypes, anti-phospholipid antibodies (anti-cardiolipin antibody, lupus anticoagulant and beta-2-glycoprotein) and a uterine cavity evaluation prior to attempting conception. After the evaluation was complete, those RPL patients (<43 years old) without a history of infertility and a history of prior spontaneous pregnancy, were encouraged to attempt spontaneous conception for 6 months. In those patients that opted for fertility treatments sooner, they were offered a range of options, including: IUI, COH and IVF. In addition, PGS with 24-chromosome screening and day 5 trophectoderm biopsy was offered as an option for those patients interested in aneuploidy screening during an IVF cycle.

Once the initial evaluation was completed, patients were followed for a subsequent pregnancy for a minimum of 6 months. Once the patient reported a missed menses or positive home pregnancy test, the patient came for a serum quantitative hCG level in our center. After the initial positive serum test (hCG >5mIU/mL), a repeat level was drawn 48 hours later and if rising appropriately, the patient was scheduled for a transvaginal ultrasound at 6–7 weeks of gestation. Pregnancies were then followed by weekly ultrasound until transfer of care at 10 weeks gestational age. A clinical miscarriage (CM) was defined as a loss of pregnancy after a gestational sac had been identified on ultrasound. A patient with a serum hCG level >5mIU/mL that never progressed to a gestational sac on transvaginal ultrasound was diagnosed with a biochemical pregnancy (BC) or pregnancy of unknown location (PUL). All pregnancies reported as ongoing had progressed past 20 weeks of gestation. Live births (LB) were documented by patient report and when results were not available, patients were contacted by their physician for follow-up.

### Statistical analysis

The continuous data with a normal distribution was reported as a mean value. A median value with its associated range was also reported when the data did not have a normal distribution. The unpaired student *t*-test was used to analyze the difference between the means and the Mann–Whitney test was used for the comparison of medians. Categorical data was presented as percentages and a Fisher’s exact test was used to present the differences between the two groups. A p-value of <0.05 was considered statistically significant.

## Results and discussion

A total of 190 fertile RPL patients completed the initial evaluation and were followed for a minimum of 6 months in our center to document subsequent pregnancy outcome (Additional file 1: Table S1). In the patients in our fertile cohort, 4% (n = 7) were diagnosed with anti-phospholipid antibody syndrome. Of these 7 patients, 1 elected for PGS, 1 elected for IUI and 5 patients became pregnant spontaneously. 3% (n = 5) of patients were carriers of a translocation. Of these 5 patients, 2 elected for PGS and 3 became pregnant spontaneously. Finally, 2% (n = 3) of patients were diagnosed with anatomic abnormalities of the uterine cavity. Of these 3 patients, 1 elected for IVF, 1 elected for IUI and 1 patient became pregnant spontaneously. Only the first pregnancy was reported and followed. Of the 190 patients followed, 158 had a laboratory confirmed pregnancy (beta hCG >5mIU/mL). The overall pregnancy rate (PR) at one year was 83% in this cohort.

Of the 158 patients with a positive pregnancy test, 98 occurred spontaneously and 60 occurred with fertility treatment. Although all of the patients included in this study were fertile, a large number of them did request to undergo fertility treatment immediately, rather than try to conceive spontaneously. Within the treatment group, there were 23 RPL patients who elected to use IVF-PGS for aneuploidy screening. Of the patients that used PGS, 8 required more than one cycle to achieve a pregnancy, ranging between 1 and 4 cycles.

The patients that attempted to conceive spontaneously were slightly younger than the group that opted for fertility treatments (34.5 vs. 35.6) but this age difference was not found to be statistically significant (p = 0.12). The subset of women who used PGS were even older, with an average age of 36.7 years. The women in both groups had similar gravidity (3.7 vs. 3.6), number of prior miscarriages (2.8 vs. 2.6) and body mass index (BMI). Of note, the women who opted to use fertility treatments had a significantly higher rate of prior treatment use, 15% compared to 3% in the spontaneous conception group. The patients in the fertility treatment group also had a significantly longer median time to pregnancy in prior pregnancies (3 months vs. 2 months) (Table 1).

**Table 1:** **Patient characteristics in those patients who conceived within 12 months of monitoring**

	Spontaneous	All treatments	P-value
	(n = 98)	(n = 60)	
Age (mean)	34.5	35.6	0.12
Gravida (mean)	3.7	3.6	0.51
Prior miscarriages (mean)	2.8	2.6	0.18
BMI (mean)	23.8	23.2	0.36
TTP in past pregnancies (median, range)	2.0 (1–12)	3.0 (1–14)	0.03^a^
Percent of prior pregnancies using infertility treatment	3%	15%	0.03^a^

^a^Statistically significant for p < 0.05.

In those pregnancies that were conceived spontaneously, the median TTP was 2 (1–10) months. The median TTP in the treatment group (excluding PGS) was significantly longer at 4 (1–12) months (p < 0.03). When we subcategorized the treatment groups, the median TTP was 3 (1–9) months for COH with IUI and 4 (1–12) months for IVF. In the PGS group, the median TTP was 5.0 (2–10) months, which is significantly longer than those pregnancies conceived spontaneously (p < 0.01). Overall, there was a statistically significant increase in time to next pregnancy when fertile RPL patients chose to use fertility treatments (especially IVF-PGS) rather than attempt conception spontaneously.

In those patients who conceived spontaneously, 88% did so within in 6 months. In the patients that achieved pregnancy with fertility treatments (excluding PGS), 84% did so within 6 months. It took those patients who conceived with PGS significantly longer to achieve pregnancy, only 70% were pregnant within 6 months (p < 0.05).

The LB/ongoing pregnancy rate was similar between the three groups, 77% in spontaneous conception group, 73% in the treatment group and 78% in the PGS group. The CM rate was also similar between the groups, 18% in the spontaneous conception group, 16% for the treatment group and 13% in the PGS group. There was also no difference in the BC and PUL rate, 6% in the spontaneous pregnancy group, 11% in the fertility treatment group and 9% in the PGS group (Table 2).

**Table 2:** **Results of patients who conceived within 12 months of monitoring**

	Spontaneous	Treatments,	p-value	PGS only	p-value
	Pregnancies	Excluding PGS	(vs. Sp)		(vs. Sp)
	(n = 98)	(n = 37)		(n = 23)	
% of pregnancies conceived within 6 months	88%	84%	0.58	70%	0.05^a^
TTP (mean) and (median, range)	3.3 +/- 2.4	4.3 +/- 2.8	0.03^a^	5.4 +/- 3.0	0.01^a^
	2.0 (1–10)	4.0 (1–12)	0.03^a^	5.0 (2–10)	0.01^a^
LB/ongoing	77%	73%	0.65	78%	1.0
CM	18%	16%	1.0	13%	0.76
BC or PUL	6%	11%	0.46	0%	0.65

^a^Statistically significant for p < 0.05.

In young fertile RPL patients, there does not appear to be clinical benefit to proceeding directly to fertility treatments to reduce the time to next pregnancy, increase the chance of a LB or decrease the chance of a subsequent miscarriage. Those RPL patients who conceived spontaneously in the past and were followed for 6 months had an overall PR of 83% within one year. Of those RPL patients that got pregnant, 84% did so within 6 months. Using any form of fertility treatment did not decrease this time to pregnancy, it actually increased the time significantly. This increase in time may be due to several factors. Patients in the treatment group were slightly older and the preparation or set up time for an IUI or IVF cycle may take 1–2 months. Additionally, given this is a retrospective review, it is impossible to say if patients tried to conceive spontaneously before starting fertility treatment. If patients who were initially considering treatment conceived spontaneously on their own, these patients would have increased the number of early spontaneous conceptions that were reported.

The LB rate is also similar between the spontaneous conception and fertility treatment groups, confirming that most fertility treatments do not have a role in the treatment of RPL. Within the group of RPL patients that became pregnant spontaneously only 18% had a CM, which was similar to the RPL patients that used fertility treatment. The high spontaneous conception rate in just 6 months of observation, justifies delaying fertility treatment for at least this period of time in this population. Our findings on PGS are similar to those pregnancies from spontaneous conception and other fertility treatments. However, due to the small number of patients that used it, we cannot make any general conclusions about the utility of PGS in an RPL population. We can report that those patients that used PGS did not appear to get pregnant any faster than those that attempted to conceive spontaneously. This finding supports the ASRM guideline that PGS does not appear to be beneficial for patients with RPL and is therefore not recommended as a treatment modality in this population. In addition, the patients that used PGS did not appear to have better pregnancy outcomes than those women who conceived spontaneously, with a similar LB and CM rate. Future studies on the management of RPL patients will benefit from a prospective study design to enable randomization of patients to various treatment groups as well as subgroup analysis of results by age group to further elucidate the possible effects of differences in maternal age on study outcomes. In addition, comparing fertile versus infertile RPL patients is likely to produce differing clinical outcomes with expectant management versus infertility treatment, and may better define a role for IVF in the treatment of patients with RPL.

## Conclusion

Our study indicates that fertility treatments in a fertile RPL cohort are unlikely to significantly improve the chances of achieving an ongoing pregnancy within 6 months of a negative work-up. These findings can be used to counsel patients with RPL regarding high rates of fertility and the likelihood of success with expectant management.

## Availability of supporting data

A data set supporting the results of this article is included within the additional files.

## Electronic supplementary material

Additional file 1: Table S1: Data set supporting the results of this article. (XLSX 57 KB)

## Abbreviations

RPL: Recurrent pregnancy loss
TTP: Time to pregnancy
PGS: Preimplantation genetic screening
IUI: Intrauterine insemination
COH: Controlled ovarian hyperstimulation
IVF: In vitro fertilization
PR: Pregnancy rate
LB: Live birth
CM: Clinical miscarriage
BC: Biochemical pregnancy
PUL: Pregnancy of unknown location.
